# Suture of Nerves, and Alternative Methods of Treatment by Transplantation of Tendon

**Published:** 1918

**Authors:** 


***Suture of Nerves, and Alternative Methods of Treatment by Transplantation of Tendon.*** By Lieut.-Colonel ROBERT JONES, Inspector of Military Orthopedics, Army Medical Service, *Brit. Med. Jour.*, May 6 and 13, 1916.

With regard to late suture of the nerve, certain general principles must be borne in mind :

1. The correction of contractures of skin or muscle and all the anatomical constituents, from skin to bone, on the side of the abnormal direction the contracture takes.

2. When possible, the freeing of joints from all adhesions and the restoration of the mobility of the joint in all cases where ankylosis is threatened.

3. The maintenance of the paralysed muscles in a position of relaxation throughout the period of recovery.

4. The practice of massage during recovery, but without once allowing the relaxed muscles to be stretched.

Before any operations are performed affecting the mobility of a joint, every use should be made of available muscle power. Neglect of this precaution may produce the unexpected recovery of muscles which were ignored because assumed to be paralysed. It is only possible to make quite certain by relaxing the muscle and thus putting it into the position most favorable to recovery for a sufficient length of time (6 months).

The first stage of treatment is the correction of existing deformity. When this has been done the limb should be kept immovable until the ligaments, muscles, and even bone have become of normal length and shape. The continuity of treatment must be maintained or a relapse will result. This point is fundamental.

Tendon transplantation has frequently been performed with success in cases in which isolated nerves have been destroyed. Its object is to improve or restore muscular balance; it is not justified unless it improves function. The following transplantation operations may be recommended in various injuries.

For irreparable injury of the musculo-spiral nerve, *a*) the flexor carpi radialis and the flexor carpi ulnaris can be transplanted into the paralysed extensor of thumb and fingers; and *b*) the pronator radii teres may, in addition, be affixed to the two radial extensors.

For great damage to the median and ulnar nerves, operations on tendons alternative to those on the nerves will be very rarely required as compared with those on the external popliteal and the musculo-spiral, for the reason that by means of flexion of the elbow a gap of two or three inches in the median may be closed up; by flexing the elbow and displacing the ulnar to the front a similar space in this nerve can be obliterated. End-to-end suture, therefore, is much more easily secured in these two nerves than in the case of the musculo-spiral and external popliteal. The transplantation operation consists in *a*) inserting the outer tendons of the flexor sublimis into the inner tendons of the flexor profundus digitorum, and *b*) inserting the inner tendons of the flexor sublimis into the tendon of the flexor carpi ulnaris. The extensor carpi radialis is attached to the flexor longus pollicis.

The operation of transplantation in complete paralysis of the ulnar consists in attaching the two inner tendons of the flexor profundus to the two outer, and inserting the palmaris longus into the tendon of the flexor carpi ulnaris.

After operations for musculo-spiral paralysis the hand should be kept dorsiflexed until recovery of the muscle is complete. Even after the patient has learned to use his hand a dorsiflexion splint should be worn at night.

For paralysis of the crural nerve the sartorius and biceps may be transplanted into the patella.

In paralysis of muscles supplied by the external popliteal nerve there is not much scope for effective tendon transplantation; for an injury paralysing the two peronei muscles but leaving the anterior tibial nerve intact, transplantation of the insertion of the tibialis anticus into the dorsum of the cuboid or into the base of the fifth metatarsal replaces the loss of the evertors and restores the balance of the foot. In cases of more extensive paralysis there is not sufficient muscle power remaining for it to be effectually distributed, and tendon fixation is then the best operative procedure, for it establishes a firm barrier against drop-foot and yet allows useful mobility.

In injuries to the sciatic trunk, when the whole nerve has been divided high up in the thigh and there is total loss of power below the knee, the patient can walk quite well in a jointed caliper splint with a filling inside the boot to keep the foot at right angles. Another useful plan is to fit a jointed knee cage with a spring and a right-angled support for the ankle, thus making the paralysed distal part of the leg into a species of artificial limb. The idea of rushing to amputation of a limb merely because the sciatic nerve is destroyed is too horrible to be contemplated, and actual experience has proved that the nutritive disturbances which should theoretically take place in the foot either do not occur or, at worst, are not so serious as expected.

